# Murine Polyomavirus Virus-Like Particles Carrying Full-Length Human PSA Protect BALB/c Mice from Outgrowth of a PSA Expressing Tumor

**DOI:** 10.1371/journal.pone.0023828

**Published:** 2011-08-17

**Authors:** Mathilda Eriksson, Kalle Andreasson, Joachim Weidmann, Kajsa Lundberg, Karin Tegerstedt, Tina Dalianis, Torbjörn Ramqvist

**Affiliations:** Department of Oncology-Pathology, Karolinska Institutet, Stockholm, Sweden; Federal University of São Paulo, Brazil

## Abstract

Virus-like particles (VLPs) consist of capsid proteins from viruses and have been shown to be usable as carriers of protein and peptide antigens for immune therapy. In this study, we have produced and assayed murine polyomavirus (MPyV) VLPs carrying the entire human Prostate Specific Antigen (PSA) (PSA-MPyVLPs) for their potential use for immune therapy in a mouse model system. BALB/c mice immunized with PSA-MPyVLPs were only marginally protected against outgrowth of a PSA-expressing tumor. To improve protection, PSA-MPyVLPs were co-injected with adjuvant CpG, either alone or loaded onto murine dendritic cells (DCs). Immunization with PSA-MPyVLPs loaded onto DCs in the presence of CpG was shown to efficiently protect mice from tumor outgrowth. In addition, cellular and humoral immune responses after immunization were examined. PSA-specific CD4^+^ and CD8^+^ cells were demonstrated, but no PSA-specific IgG antibodies. Vaccination with DCs loaded with PSA-MPyVLPs induced an eight-fold lower titre of anti-VLP antibodies than vaccination with PSA-MPyVLPs alone. In conclusion, immunization of BALB/c mice with PSA-MPyVLPs, loaded onto DCs and co-injected with CpG, induces an efficient PSA-specific tumor protective immune response, including both CD4^+^ and CD8^+^ cells with a low induction of anti-VLP antibodies.

## Introduction

Prostate cancer is the second most frequently diagnosed cancer in men globally, with 782 600 new cases and an estimated 254 000 deaths in 2007 [1]. If the cancer is detected early and is localized within the prostatic capsule, it can be cured by surgery or radiotherapy. However, the prognosis is often poor if metastasis has already occurred at the time of diagnosis, with an average survival of 2.5 years [2], [3]. The mainstay of therapy for metastatic prostate cancer is androgen ablation accomplished by either androgen-antagonistic agents or castration [4]. Although androgen withdrawal prolongs the period free of disease progression, prostate tumor cells eventually become independent of androgen, resulting in relapse [5], [6]. Despite major advances in the treatment of prostate cancer during the last decades, current therapies are usually debilitating, causing impotence and incontinence resulting in low quality of life for the patient. Consequently, there is a need for new and less damaging treatments and immunotherapy might represent one such strategy.

Current immunotherapeutic strategies against prostate cancer include administration of antibodies [7]–[9] and different kinds of cancer vaccines, for example administration of peptides derived from prostate antigen proteins [10], whole tumor cells [11], dendritic cells (DCs) loaded with peptides [12] or tumor cell lysates [13], and DNA encoding human Prostate Specific Antigen (PSA) [14]. Some of these developments are promising. However, it is probable that additional approaches are necessary to combat metastatic prostate cancer.

PSA is a chymotrypsin-like serine protease that has a highly restricted tissue distribution and is expressed in the epithelial cells of the prostate gland, thus in the same cell type from which most prostate tumors arise [15]. Its expression is regulated by androgen, and it is present at extremely low levels in the circulation of adult men [16]. Most prostate tumors, even the poorly differentiated ones, continue to express and release PSA [17]. Thus, PSA is widely used as a serum marker for prostate cancer [18]. The almost exclusive tumor specific expression of PSA makes it a potential target antigen for anti-tumor cytotoxic T lymphocytes (CTLs). In addition, detection of anti-PSA antibodies and circulating CD8^+^ T cells in patients with advanced prostate cancer indicates that PSA can be the target of an autoimmune response and that tolerance to PSA is not absolute [19]–[23].

Virus-like particles (VLPs) are spontaneously self-assembled capsid proteins from viruses such as papillomavirus, rotavirus and polyomavirus [24]–[26]. These particles have been shown to be exploitable for vaccination against viral infection, where the best-known examples are the VLP based vaccines against various types of Human Papilloma Virus [27], [28]. In addition, VLPs have also been used as carriers of foreign genetic material or protein and peptide antigens for gene and immune therapy. More specifically, chimeric VLPs, carrying tumor antigens fused to capsid proteins, have successfully been used to prevent outgrowth of tumors in several mouse models [29]–[31]. Furthermore, our group has previously shown that VLPs from murine polyomavirus (MPyVLPs) carrying the breast cancer antigen Her2 (Her2-MPyVLPs) can protect mice from outgrowth of the Her2-expressing murine tumor cell D2F2/E2, as well as spontaneous tumor formation in transgenic BALB-neuT mice [29]. In the same system, it was also shown that co-injection of Her2-MPyVLPs with adjuvant CpG, or loading of Her2-MPyVLPs onto DCs could improve the efficiency of these vaccines [32]–[34].

In this study, we produced MPyVLPs carrying human full-length PSA (PSA-MPyVLPs) and explored the possibility to use these VLPs in an experimental model to immunize BALB/c mice and protect them from outgrowth of the PSA-expressing tumor D2F2/PSA. To enhance protection, we also co-injected PSA-MPyVLPs with adjuvant CpG, in combination with loading them onto murine DCs *in vitro* before immunization. Finally, cellular and humoral immune responses were examined after the different immunization protocols.

## Materials and Methods

### Ethics Statement

The animal experiments in this study were conducted according to Ethical permissions N357/07 and N351/09 from Stockholms Norra Djurförsöksetiska Nämnd, Sweden. All efforts were made to minimize suffering.

### Construction, purification and characterization of MPyVLPs and PSA-MPyVLPs

The VP1 gene from MPyV (from pPYwt [35]) was cloned under the p10 promoter in the baculovirus transfer vector pAcDB3 (BD Biosciences Pharmingen, San Diego, CA) as described earlier [29]. The PSA gene (from pVAX-PSA [14]) was fused via an alanine linker to the 306 C-terminal nucleotides of VP2/VP3 from MPyV and cloned under the polyhedrin promoter in the same plasmid. Recombinant baculoviruses were produced and generation and purification of VLPs from these baculoviruses were performed as described earlier [29], here using a PSA specific immunoblot with a polyclonal rabbit anti-human PSA antibody (DakoCytomation, Glostrup, Denmark). MPyVLPs were produced as described previously [29]. The morphology of the purified VLPs was analysed by electron microscopy as described earlier [36]. The approximate number of VP2/3-PSA molecules per PSA-MPyVLP was estimated using an immunoblot including a serial dilution of human recombinant PSA (Fitzgerald Industries International, North Acton, USA).

### Mice

Female BALB/c mice, aged 6–10 weeks, were bred and maintained at the Dept. of Microbiology, Tumor and Cell Biology, Karolinska Institutet.

### Cell line

The murine mammary cell line D2F2 (H-2^d^) was co-transfected with pVAX-PSA [14] expressing full length human PSA and pcDNA3 expressing the neomycin resistance gene, using Lipofectamine 2000 reagent (Invitrogen, Carlsbad, CA, USA), according to the manufacturer's instructions, to obtain a PSA positive H-2^d^ cell line. Cells were selected, cloned and analysed for expression of PSA by immunoblot. One clone (D2F2/PSA) with a strong PSA expression was selected for use for *in vivo* experiments. D2F2/PSA cells were cultured as previously described for D2F2/E2 [32].

### Recombinant proteins and peptides

For *in vitro* experiments the following proteins and peptides were used: human recombinant PSA (Fitzgerald Industries International, North Acton, USA), Human Serum Albumin (HSA) (Octapharma AB, Stockholm, Sweden), Phytohaemagglutinin (PHA) (Sigma-Aldrich Sweden AB, Stockholm, Sweden), PSA immunodominant CD4-restricted peptide PSA_238-253_ (ERPSLYTKVVHYRKWI), and PSA immunodominant CD8-restricted peptide PSA_188-197_ (HPQKVTKFML) [37] (both from ProImmune, Oxford, UK).

As a negative control the LCMV-derived peptide NP_118-126_ (RPQASGVYM) (Genscript Corporation, New Jersey, USA) was used. As a positive control phorbol myristate (PMA) together with ionomycin (IO) (Sigma-Aldrich Sweden AB, Stockholm, Sweden) was used.

### Purification of murine DCs and loading with VLPs

Murine spleen derived DCs were isolated using CD11c MicroBeads (Miltenyi Biotec, Bergisch Gladbach, Germany) and loaded with VLPs as previously described [32]. Briefly, murine spleens were digested with 2 mg/ml collagenase type D (Roche Diagnostics), labelled with anti-CD11c magnetic beads and enriched on MACS columns according to manufacturer's protocol. CD11c^+^ DCs, 2×10^5^ per animal to be vaccinated, were then incubated with human recombinant PSA (2 µg), MPyVLPs or PSA-MPyVLPs (50 µg) one hour on ice, and washed twice.

### Immunization and tumor cell challenge

For *in vivo* tumor rejection tests ten BALB/c mice per group were immunized with a single subcutaneous injection in the flank of 50 µg PSA-MPyVLPs, or MPyVLPs as a negative control, or 2 µg human recombinant PSA (corresponding to the approximate amount of PSA in 50 µg PSA-MPyVLPs), with or without 50 µg CpG 1826 (InvivoGen, San Diego, CA, USA) as adjuvant, and when indicated loaded onto 2×10^5^ DCs. Two weeks later mice were injected with 5×10^4^ D2F2/PSA cells subcutaneously in the flank. The incidence and growth of tumors were measured twice weekly by measuring three diameters, to establish tumor volume. Mice were sacrificed when the tumor diameter reached 10 mm in any dimension.

For *in vitro* assays three mice per group were immunized in the same way as above and sacrificed one week later to harvest the splenocytes.

### Enzyme-Linked Immunosorbent Spot Assay (ELISpot)

An IFNγ ELISpot was performed according to the manufacturer's instructions (Mabtech, Nacka, Sweden). Briefly, splenocytes (1.2×10^5^) were cultured in triplicates for 20 hours in ELISpot plates coated with an anti-mouse IFNγ antibody, alone or together with 5 µg/ml recombinant PSA, PSA_238-253_, PSA_188-197_, or NP_118-126_ as negative control, or 25 ng/ml PMA together with 250 ng/ml IO as positive control. Spots were detected and counted in an ELISpot reader (AID, Strassberg, Germany).

### Intracellular IFNγ staining and proliferation assay

Analysis of intracellular IFNγ production in PSA-specific CD4^+^ and CD8^+^ cells after immunization was performed as previously described [38]. In short, splenocytes from mice were stimulated with 5 µg/ml PSA_238-253_, PSA_188-197_, NP_118-126_ as negative control, or 25 ng/ml PMA together with 250 ng/ml IO as positive control, and incubated at 37°C for 5 days. Thereafter, splenocytes were collected and subjected to a second stimulation with the same stimuli as above for 4 hours. Brefeldin A was added after 2 hours, followed by surface staining of CD4 or CD8 and intracellular staining for IFNγ. Cells were then analysed using flow cytometry.

Analysis of proliferation of CD4^+^ and CD8^+^ cells after immunization was performed using the APC BrdU Flow Kit according to the manufacturer's protocol (BD Biosciences Pharmingen, San Diego, CA, USA). Briefly, splenocytes were cultured together with 5 µg/ml recombinant PSA, 2.5 µg/ml HSA as negative control, or 10 µg/ml PHA as positive control, and incubated at 37°C for 72 hours. 1 µM BrdU (thymidine analogue) was added to all cells and incubated at 37°C overnight. Cells were thereafter stained for intracellular BrdU and surface CD4 or CD8, and analysed using flow cytometry.

### Flow cytometry

Flow cytometry was performed on a FACSCalibur with CellQuest Pro software (BD Biosciences Pharmingen, San Diego, CA, USA) using directly conjugated mAbs against the following markers: PE anti-mouse IFNγ, FITC anti-mouse CD4, PE anti-mouse CD4, FITC anti-mouse CD8, APC anti-mouse CD8 and APC anti-BrdU (all from BD Biosciences Pharmingen, San Diego, CA, USA).

### Enzyme-Linked ImmunoSorbent Assay (ELISA)

Mouse sera, collected 13 days after immunization from tail veins, were analysed for presence of anti-VP1 and anti-PSA IgG antibodies using an ELISA as previously described [32]. Briefly, microtiter plates were coated with 5 µg/ml MPyVLPs or recombinant PSA. Sera were added, as well as a mouse anti-human PSA antibody (DakoCytomation, Glostrup, Denmark) as positive control for the PSA-ELISA, and after one-hour incubation, and washing, secondary alkaline phosphatase-conjugated goat anti-mouse IgG (Sigma-Aldrich Sweden AB, Stockholm, Sweden) was added for one hour. Plates were developed with nitrophenylphosphate tablets (Sigma-Aldrich Sweden AB, Stockholm, Sweden) and absorbance was measured at 405 nm.

### Statistical analyses

Differences in tumor takes were analysed by Fisher's exact two-tailed test. Student's t-test was used to analyse differences in *in vitro* stimulation, proliferation, ELISpot and ELISA data.

## Results

### Construction and characterization of MPyVLPs carrying a VP2/3-PSA fusion protein

**Figure 1:**
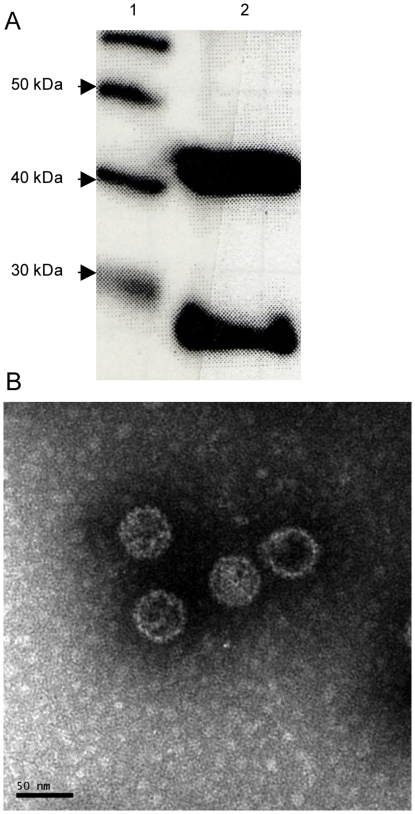
Characterization of PSA-MPyVLPs. (A) Immunoblot using a polyclonal rabbit anti-human PSA antibody showing the VP2/3-PSA fusion protein as a band of approximately 41 kDa. We also see a smaller fusion protein of approximately 25 kDa, probably due to alternative splicing or post-translational proteolytic cleavage. Lane 1: Protein Ladder, Lane 2: PSA-MPyVLPs. (B) Electron microscopy showing PSA-MPyVLPs with an approximate diameter of 45 nm.

Virus-like particles consisting of full-length MPyV VP1 and a fusion protein between the complete PSA protein and the 102 C-terminal amino acids of the VP2/VP3 protein (denoted PSA-MPyVLPs) were produced using a baculovirus transfection vector system. PSA-MPyVLPs were analysed on immunoblot (Figure 1A) and demonstrated to contain the VP2/3-PSA fusion protein besides VP1. The purified PSA-MPyVLPs also turned out to contain a smaller protein, also recognized by the anti-PSA antibody, with a size of approximately 25 kDa (Figure 1A). This protein most likely represents a shorter version of the VP2/3-PSA protein produced either by alternative splicing or post-translational proteolytic cleavage. Electron microscopy showed VLPs with a diameter of approximately 45 nm (Figure 1B). The number of VP2/3-PSA molecules per PSA-MPyVLP was estimated to around 20 by comparing band strength to a serial dilution of human recombinant PSA on an immunoblot (data not shown). Thus when immunizing mice with around 50 µg of PSA-MPyVLPs, approximately 2 µg of PSA protein was included.

### DCs loaded with PSA-MPyVLPs and co-injected with CpG protect BALB/c mice against outgrowth of a PSA-expressing tumor

**Figure 2:**
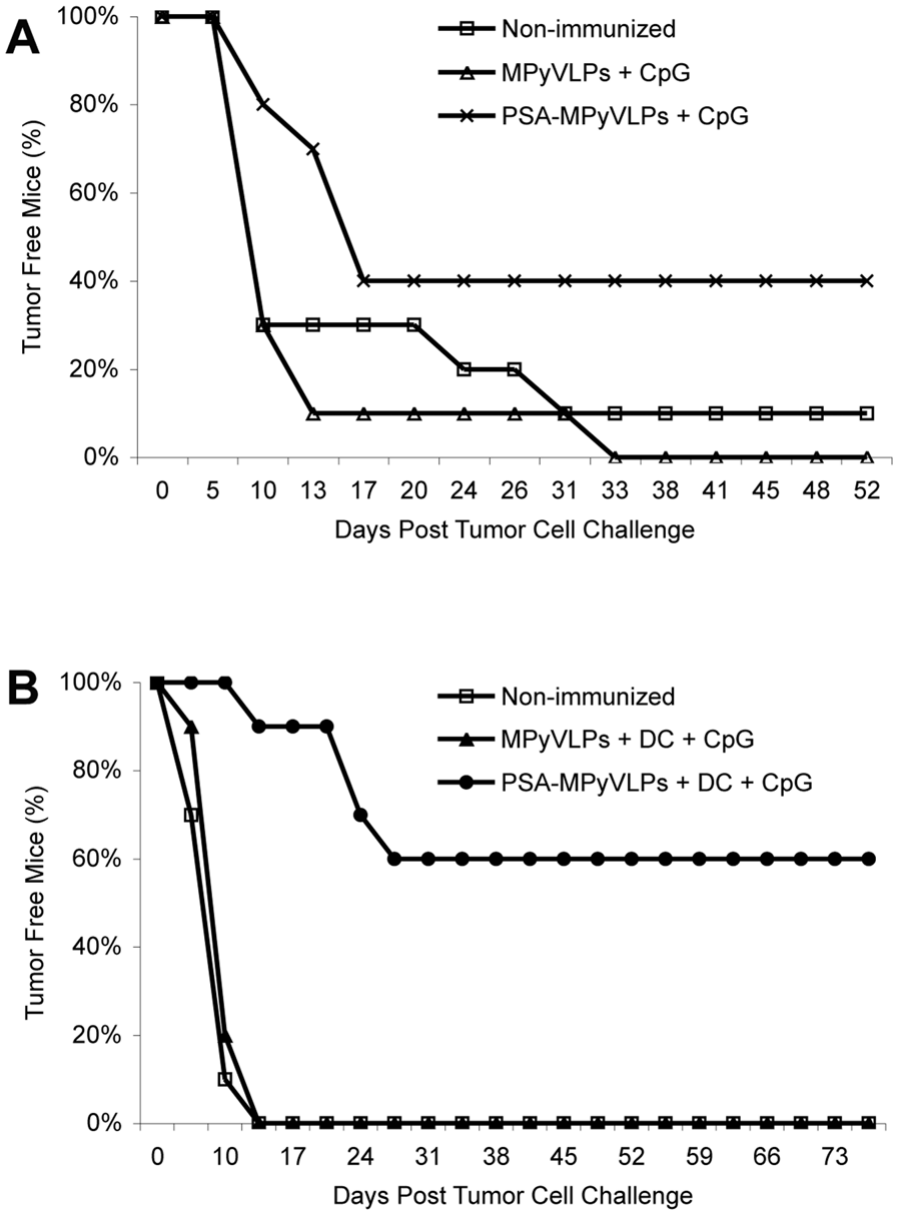
Tumor outgrowth in BALB/c mice. Illustration of experiments 3 and 6, (A) and (B) respectively, according to Table 1.

To test if PSA-MPyVLPs could induce protective immunity, seven experiments with ten mice per group, receiving different vaccination combinations, were performed and the data are summarized in Table 1, while two representative experiments are shown in Figure 2. In all experiments each mouse was only vaccinated once. Two weeks after immunization mice were challenged with a lethal dose of PSA-positive D2F2/PSA cells and monitored for tumor outgrowth.

**Table 1:** Tumor outgrowth in BALB/c mice.

Immunogen	Exp.1	Exp.2	Exp.3	Exp.4	Exp.5	Exp.6	Exp.7	Total takes	%	p value^a^
Non-immunized	10/10	9/10	9/10	5/10	5/10	10/10	7/10	55/70	79	ref.
MPyVLPs + CpG			10/10					10/10	100	ns^b^
MPyVLPs/DC + CpG						10/10	6/10	16/20	80	ns^c^
PSA/DC + CpG							4/10	4/10	40	ns^d^
PSA-MPyVLPs	6/10	7/10					2/10	15/30	50	<0.01^e^
PSA-MPyVLPs + CpG		5/10	6/10	5/10	4/10		3/10	23/50	46	<0.05^f^
PSA-MPyVLPs/DC + CpG				2/10	0/10	4/10	0/10	6/40	15	<0.001^g^

a Compared to non-immunized mice according to respective grouping

b Exp. 3,

c Exp. 6 and 7,

d Exp. 7,

e Exp. 1, 2 and 7,

f Exp. 2–5 and 7,

g Exp. 4-7.

When summarizing all *in vivo* data (Table 1), immunization with PSA-MPyVLPs alone resulted only in a weak protection against outgrowth of PSA-expressing D2F2/PSA tumor cells, with a 50% tumor take as compared to 79% in non-immunized mice (p<0.01). Co-injection of PSA-MPyVLPs and CpG resulted in a protective effect with a 46% tumor take (p<0.05), while loading of PSA-MPyVLPs onto DCs and co-injecting with CpG further reduced the tumor take to 15% (p<0.001) (Table 1). Furthermore, vaccinating with PSA-MPyVLPs loaded on DCs with CpG was significantly more efficient as compared to vaccinating with either PSA-MPyVLPs alone (p<0.01), or together with CpG (p<0.01), while there was no significant difference between the latter two (data not shown). Immunization with human recombinant PSA in combination with CpG and DCs resulted in a 40% tumor take (p = ns) (Table 1). Statistical analyses for the respective immunization groups are presented in Table 1. Protection was PSA-specific, since in the groups immunized with MPyVLPs loaded onto DCs and co-injected with CpG 80% of the mice developed tumors (p = ns) (Table 1).

The D2F2/PSA cell line was tested for PSA expression using immunoblot after passage *in vivo*, in both immunized and non-immunized mice, and was shown to still express PSA (data not shown).

### PSA-specific CD4^+^ and CD8^+^ cells are induced by PSA-MPyVLP immunization

The induction of PSA-specific CD4^+^ and CD8^+^ cells after vaccination with PSA-MPyVLPs was assayed for by using three different methods, which all were repeated at least twice. Splenocytes from immunized and non-immunized mice, stimulated with recombinant PSA or PSA peptides (CD4 or CD8-restricted), were analysed for IFNγ secretion by ELISpot, intracellular IFNγ production by flow cytometry, or for proliferation by BrdU-incorporation assay.

**Figure 3:**
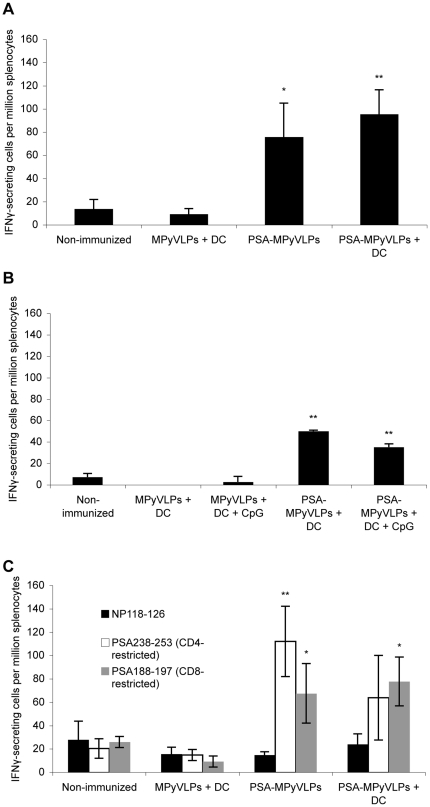
IFNγ-secretion from PSA-specific cells. ELISpot analysis. (A and B) Splenocytes stimulated with recombinant PSA. Significantly more PSA-specific secretion of IFNγ was induced after immunization with PSA-MPyVLPs alone (p <0.05), PSA-MPyVLPs on DCs (p <0.01), and PSA-MPyVLPs on DCs with CpG (p <0.01) as compared to non-immunized mice. (C) Splenocytes stimulated with PSA immunodominant CD4- or CD8 restricted peptides. PSA-MPyVLP immunization induced both PSA-specific CD4^+^ (p <0.01) and CD8^+^ cells (p <0.05). Loading of PSA-MPyVLPs onto DCs resulted in production of PSA-specific CD8^+^ cells (p <0.05). Shown are representative bar-graphs from three experiments with three mice in each group. Values from non-stimulated cells have been subtracted. Error bars represent S.D. p values calculated using Student's t-test (*  =  p<0.05, **  =  p<0.01).

When assayed by ELISpot using recombinant PSA, significantly more PSA-specific secretion of IFNγ was induced after immunization with PSA-MPyVLPs alone (p<0.05), PSA-MPyVLPs on DCs (p<0.01), and PSA-MPyVLPs on DCs with CpG (p<0.01) as compared to splenocytes from non-immunized mice or mice immunized with MPyVLPs (Figure 3A and B). When assaying by ELISpot using CD4- and CD8 specific peptides, it was also possible to show that both CD4^+^ (p<0.01) and CD8^+^ (p<0.05) PSA-specific cells were observed after PSA-MPyVLP immunization (Figure 3C). However, after immunization with PSA-MPyVLPs loaded DCs a significant production of PSA-specific CD8^+^ cells (p<0.05) was observed, while the PSA-specific CD4^+^ response was weaker (Figure 3C).

**Figure 4:**
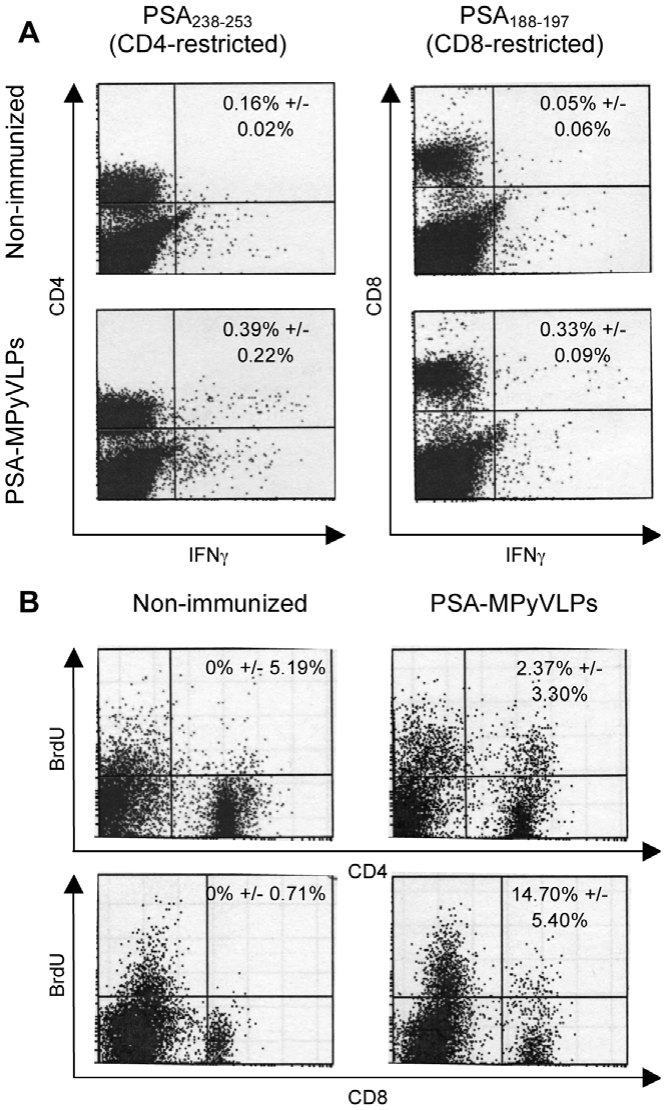
IFNγ production and proliferation of PSA-specific CD4^+^ and CD8^+^ cells. (A) Intracellular IFNγ staining. PSA-MPyVLP immunization induced significantly more PSA-specific CD8^+^ cells compared to non-immunized mice (p <0.05) after a five day *in vitro* stimulation period. Shown are representative flow cytometric dot-plots from two experiments with three mice per group. Numbers in upper right quadrants represent mean percentage IFNγ-producing cells out of all CD4^+^ or CD8^+^ cells +/− S.D. for both experiments performed. Values from cells stimulated with control peptide NP_118-126_ have been subtracted. (B) *In vitro* proliferation assay. PSA-MPyVLP immunization induced significant proliferation of PSA-specific CD4^+^ cells compared to non-immunized mice (p <0.01). Shown are representative flow cytometric dot-plots from two experiments with three mice per group. Numbers in upper right quadrants represent mean percentage CD4^+^ or CD8^+^ cells out of all proliferating cells +/− S.D. for both experiments performed. Values from cells stimulated with control protein HSA have been subtracted. p values calculated using Student's t-test (*  =  p<0.05).

PSA-MPyVLP immunization was also shown to induce significantly more CD8^+^ IFNγ-producing cells (p<0.05) when analysed by flow cytometry after a five day *in vitro* stimulation period (Figure 4A), as well as significantly more proliferation of PSA-specific CD4^+^ cells (p<0.01) compared to non-immunized mice (Figure 4B).

### PSA-MPyVLP immunization does not induce production of PSA-specific IgG antibodies

To assay for the induction of PSA-specific IgG antibodies, sera from PSA-MPyVLP immunized mice were analysed by an ELISA coated with recombinant PSA. No anti-PSA IgG antibodies were detected in any of the immunized or non-immunized mice (data not shown).

### Vaccination with DCs loaded with PSA-MPyVLPs induces lower titres of anti-VP1 antibodies than vaccination with PSA-MPyVLPs alone

**Figure 5:**
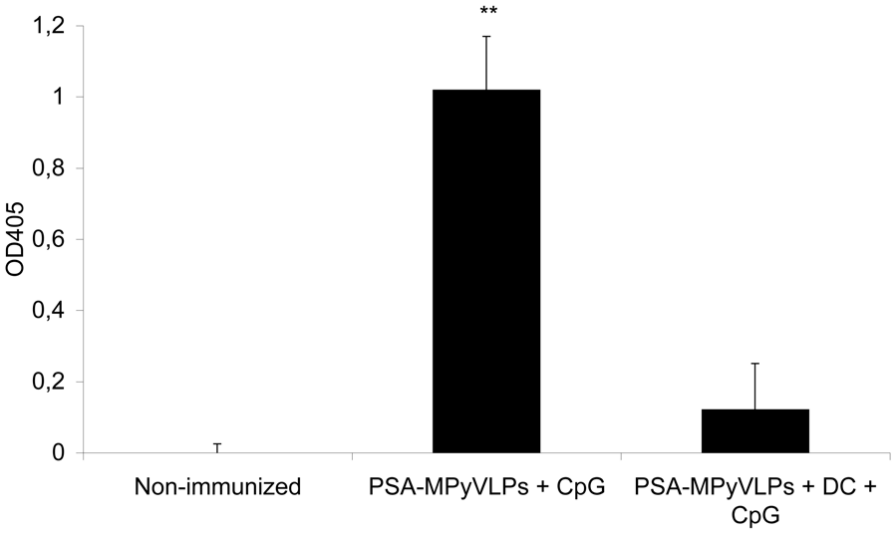
Anti-VP1 antibodies in sera. Loading of PSA-MPyVLPs onto DCs decreased the anti-VP1 IgG antibody response 8-fold compared to immunization with PSA-MPyVLPs alone (p <0.0001). Error bars represent S.D. p values calculated using Student's t-test (** p<0.01).

To detect anti-VP1 IgG antibodies, an ELISA coated with MPyVLPs was performed on sera from vaccinated and control mice. Immunization with PSA-MPyVLPs and CpG induced 8-fold higher titres of anti-VP1 IgG compared to immunization with the same type of VLPs loaded onto DCs and injected with CpG (p<0.0001) (Figure 5).

## Discussion

In this study the possibility to produce MPyVLPs carrying human full-length PSA and the ability to use these PSA-MPyVLPs together with CpG and DCs to immunize against a PSA positive tumor (D2F2/PSA) in BALB/c mice was demonstrated. In addition, cellular and humoral immune responses after different immunization protocols were examined *in vitro*.

PSA-MPyVLPs were demonstrated both to have an intact capsid and to contain the VP2/3-PSA fusion protein. These results are in line with previous results showing that it is possible to produce chimeric MPyVLPs carrying large proteins, without disrupting the capsid structure, which makes them useful as immunogens [29], [32].

When PSA-MPyVLPs were used alone, or together with CpG for immunization, there was a decrease in outgrowth of D2F2/PSA. In comparison to the previously very efficient immunization with another tumor antigen (Her2), with Her2-MPyVLPs [29], the obtained effect after immunizing with PSA-MPyVLPs alone or with CpG was clearly weaker. The reason for this difference could be due to properties of the antigens, e.g. PSA is secreted from the cells, while Her2 is a surface antigen. However, when loading PSA-MPyVLPs onto DCs as well as adding CpG, the immunization efficiency improved, which was also in line with previous findings for Her2-MPyVLPs [32]. It has been suggested that loading of VLPs onto DCs could induce maturation of the DCs. However, in spite of extensive efforts we have never demonstrated the upregulation of maturation markers, such as MHC II, CD80 or CD86, when murine DCs were incubated with Her2-MPyVLPs *in vitro* [32].

IFNγ-secreting cells were activated after immunization with PSA-MPyVLPs, as demonstrated by ELISpot where splenocytes were stimulated with recombinant PSA, and loading of PSA-MPyVLPs onto DCs improved the response as compared to immunization with PSA-MPyVLPs alone. However, the response measured with ELISpot after immunization with PSA-MPyVLPs on DCs injected with CpG was weaker than the response after immunization without CpG. This seems rather surprising, but has been demonstrated previously, both by our group (unpublished data) and by Lubaroff et al [39]. The latter reported in 2006 that co-administration of an adenovirus-PSA vaccine and CpG ODN increased tumor protection in a mouse model of prostate cancer and at the same time decreased cytotoxic T cell activity *in vitro*.

By ELISpot, stimulating splenocytes with PSA CD4- or CD8 restricted peptides, we could also demonstrate that immunization with PSA-MPyVLPs induced production of both CD4^+^ and CD8^+^ IFNγ-secreting cells. Loading of PSA-MPyVLPs onto DCs resulted in significant induction of PSA-specific CD8^+^ cells, compared to mice immunized with MPyVLPs on DCs as well as non-immunized mice. The fact that both CD4^+^ and CD8^+^ cells are induced was also supported by our FACS data and are in line with previous results in a similar model. In that model, BALB/c mice were depleted of different immune cells before, or during, immunization with Her2-MPyVLPs, and there we could show that CD4^+^ and CD8^+^ cells could act separately to protect mice from tumor outgrowth, while depletion of both subsets of cells completely abrogated protection [34].

PSA-specific IgG antibodies were not demonstrated after immunization with PSA-MPyVLPs. This could possibly be explained by the fact that PSA is fused to VP2/3, which are assumed to be on the inside of the VLPs, making it inaccessible to B-cells after immunization. Again this is in line with our previous studies, where anti-Her2 antibodies were not produced after immunization with Her2-MPyVLPs [29].

Furthermore, DC-vaccination with PSA-MPyVLPs reduced the antibody response to VP1 eight-fold compared to the response obtained by immunizing with PSA-MPyVLPs alone. This parallels the situation observed after immunizing with Her2-MPyVLPs alone or together with DCs, where the addition of DCs reduced the antibody response to VLPs six-fold [32]. Loading of VLPs onto DCs could thus be an advantage for prime-boost therapy, since fewer neutralizing antibodies are present.

In conclusion, immunization with PSA-MPyVLPs, loaded onto DCs and co-injected with CpG, induces an efficient immune response against PSA, which protects BALB/c mice from outgrowth of a PSA-expressing tumor. This immune response includes both CD4^+^ and CD8^+^ cells specific for PSA, in the absence of an antibody response to PSA. Furthermore, loading of VLPs onto DCs reduces the anti-VLP antibody response, favourable for prime-boost therapies.
